# Biologic therapies targeting type 2 inflammation in NSAID−exacerbated respiratory disease

**DOI:** 10.3389/fimmu.2026.1890964

**Published:** 2026-07-22

**Authors:** Piotr Szatkowski, Gülfem E. Çelik, Filip Mejza, Lucyna Mastalerz

**Affiliations:** 1 2nd Department of Internal Medicine, Jagiellonian University Medical College, Krakow, Poland; 2 Private Practitioner, Ankara, Türkiye; 3 Department of Epidemiology and Preventive Medicine, Division of Epidemiology, Jagiellonian University Medical College, Krakow, Poland

**Keywords:** aspirin hypersensitivity, biologic therapy, chronic rhinosinusitis with nasal polyps, N−ERD, type 2 inflammation

## Abstract

Nonsteroidal anti−inflammatory drug-exacerbated respiratory disease (N−ERD) is an inflammatory airway disease characterized by adult−onset asthma, chronic rhinosinusitis with nasal polyps and hypersensitivity reactions to cyclooxygenase−1 (COX−1) inhibitors. The disease is driven by dysregulated eicosanoid metabolism and sustained type 2 inflammation affecting both the upper and lower airways, resulting in severe disease and limited response to conventional therapies. In recent years, biologic agents targeting key inflammatory pathways have substantially expanded therapeutic options for patients with N−ERD. In this review, we summarize current evidence on the efficacy and mechanisms of action of approved biologic therapies, including omalizumab, anti−IL−5/IL−5Rα agents (mepolizumab, reslizumab, depemokimab, benralizumab), dupilumab and tezepelumab, with a particular focus on their effects on sinonasal disease, asthma control, aspirin tolerance, and patient−reported outcomes. Current evidence suggests that dupilumab provides the most consistent multi-domain clinical benefit, particularly in patients with refractory disease phenotypes. We further discuss emerging concepts of personalized and integrated treatment strategies combining biologics, endoscopic sinus surgery, and aspirin desensitization. Finally, we highlight unmet needs, potential biomarkers and future research directions aimed at optimizing treatment selection and improving long−term disease control in N−ERD.

## 1 Introduction

Nonsteroidal anti−inflammatory drug-exacerbated respiratory disease (N-ERD) is a heterogeneous inflammatory condition characterized by the clinical triad of asthma, chronic rhinosinusitis with nasal polyps (CRSwNP) and hypersensitivity reactions to cyclooxygenase−1 (COX−1) inhibitors, including aspirin and/or other NSAIDs (1–3). Formerly known as Widal’s or Samter’s triad, the components of N−ERD may develop sequentially over months to many years, with CRSwNP and accompanying olfactory dysfunction frequently preceding the onset of asthma (1–3). The full clinical triad of N-ERD typically evolves gradually and may take an average of 5 to 12 years to become fully established, depending on whether asthma, CRSwNP or NSAID hypersensitivity is the initial presenting manifestation (4). Respiratory reactions to COX−1 inhibitors are characterized by acute onset of nasal congestion, rhinorrhea, sneezing, nasal pruritus, ocular chemosis, and/or bronchospasm occurring within 30 to 180 minutes after drug exposure, distinguishing N−ERD from aspirin/NSAID−tolerant asthma (ATA) or CRSwNP (1–5).

N−ERD affects approximately 10% of patients with CRSwNP and 7.1% of adult asthmatics, with prevalence increasing to up to 24% in individuals with severe asthma and as high as 30% among those with severe CRSwNP (1, 2).

A well−documented history of recurrent respiratory reactions after NSAID ingestion manifesting with upper and/or lower airway symptoms in a patient with adult−onset asthma and recurrent nasal polyposis may be sufficient to establish a diagnosis of N−ERD (5). However, reliance solely on clinical history carries a risk of both under− and overdiagnosis of NSAID hypersensitivity (5). In selected cases, a controlled challenge test with aspirin or the suspected culprit drug is required to confirm the diagnosis (5, 6). The oral provocation test with aspirin or another COX−1 inhibitor remains the diagnostic gold standard, as it best mimics natural drug exposure and allows for objective assessment of respiratory reactions under controlled conditions (5, 6).

This review covers current understanding of the pathways involved in T2 inflammation, provides data on effectiveness, offers a clinical decision tree to help clinicians choose the most appropriate biologic and highlights unmet needs and areas under investigation. This review is based on a PubMed/MEDLINE search of studies on biologic therapies in N−ERD published between 2014 and 2026, using keywords such as ‘N−ERD’, ‘AERD’, ‘aspirin−exacerbated respiratory disease’ and specific biologics (dupilumab, omalizumab, mepolizumab, reslizumab benralizumab, depemokimab, tezepelumab). Studies were selected based on relevance and included randomized trials, observational studies, meta−analyses and real−world evidence. No formal systematic review methodology was applied.

### 1.1 Pathophysiology

**Figure 1:**
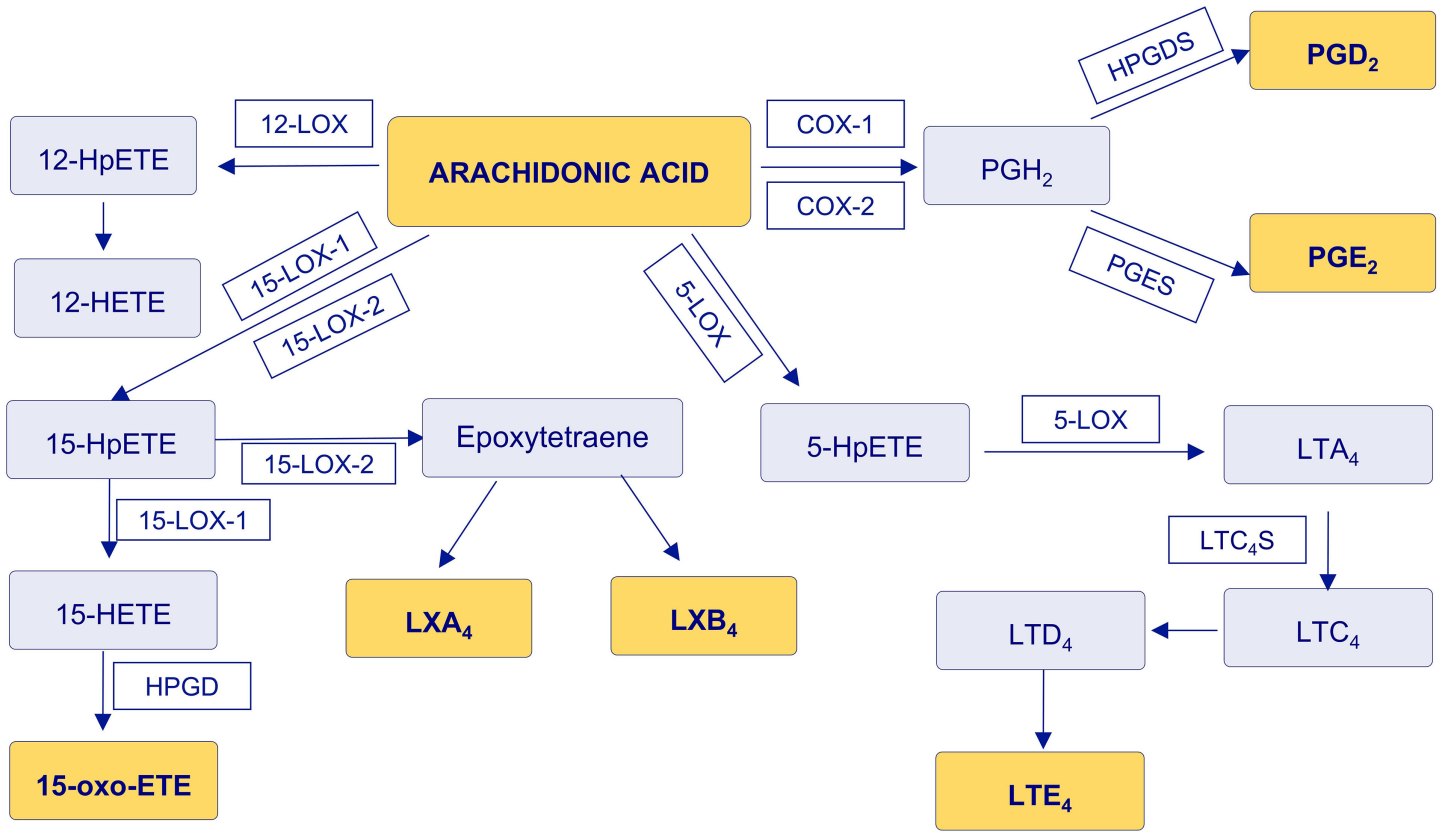
Arachidonic acid pathways. 12-HETE, 12-hydroxyeicosatetraenoic acid; 12-HpETE, 12-hydroperoxyeicosatetraenoic acid; 12-LOX, 12-lipoxygenase; 15-HETE, 15-hydroxyeicosatetraenoic acid; 15-HpETE, 15-hydroperoxyeicosatetraenoic acid; 15-LOX-1, 15-lipoxygenase type 1; 15-LOX-2, 15-lipoxygenase type 2; 15-oxo-ETE, 15-oxoeicosatetraenoic acid; 5-HpETE, 5-hydroperoxyeicosatetraenoic acid; 5-LOX, 5-lipoxygenase; COX-1, cyclooxygenase 1; COX-2, cyclooxygenase 2; HPGD, 15-hydroxyprostaglandin dehydrogenase; HPGDS, hematopoietic prostaglandin D synthase; LTA_4_, leukotriene A_4_; LTC_4_, leukotriene C_4_; LTC_4_S, leukotriene C_4_ synthase; LTD_4_, leukotriene D_4_; LTE_4_, leukotriene E_4_; LXA_4_, lipoxin A_4_; LXB_4_, lipoxin B_4_; PGD_2_, prostaglandin D_2_; PGE_2_, prostaglandin E_2_; PGES, prostaglandin E synthase; PGH_2_, prostaglandin H_2_.

N−ERD represents a distinct asthma endotype with a complex and incompletely understood pathophysiology. The disease is driven by NSAID−induced inhibition of the COX−1 enzyme, leading to dysregulated arachidonic acid metabolism characterized by excessive production of pro-inflammatory metabolites (e.g. cysteinyl leukotrienes, prostaglandin D2, 15-oxo-ETE), alongside reduced synthesis of anti-inflammatory metabolites (e.g. prostaglandin E2) (1–3, 7), see **Figure 1**.

**Figure 2:**
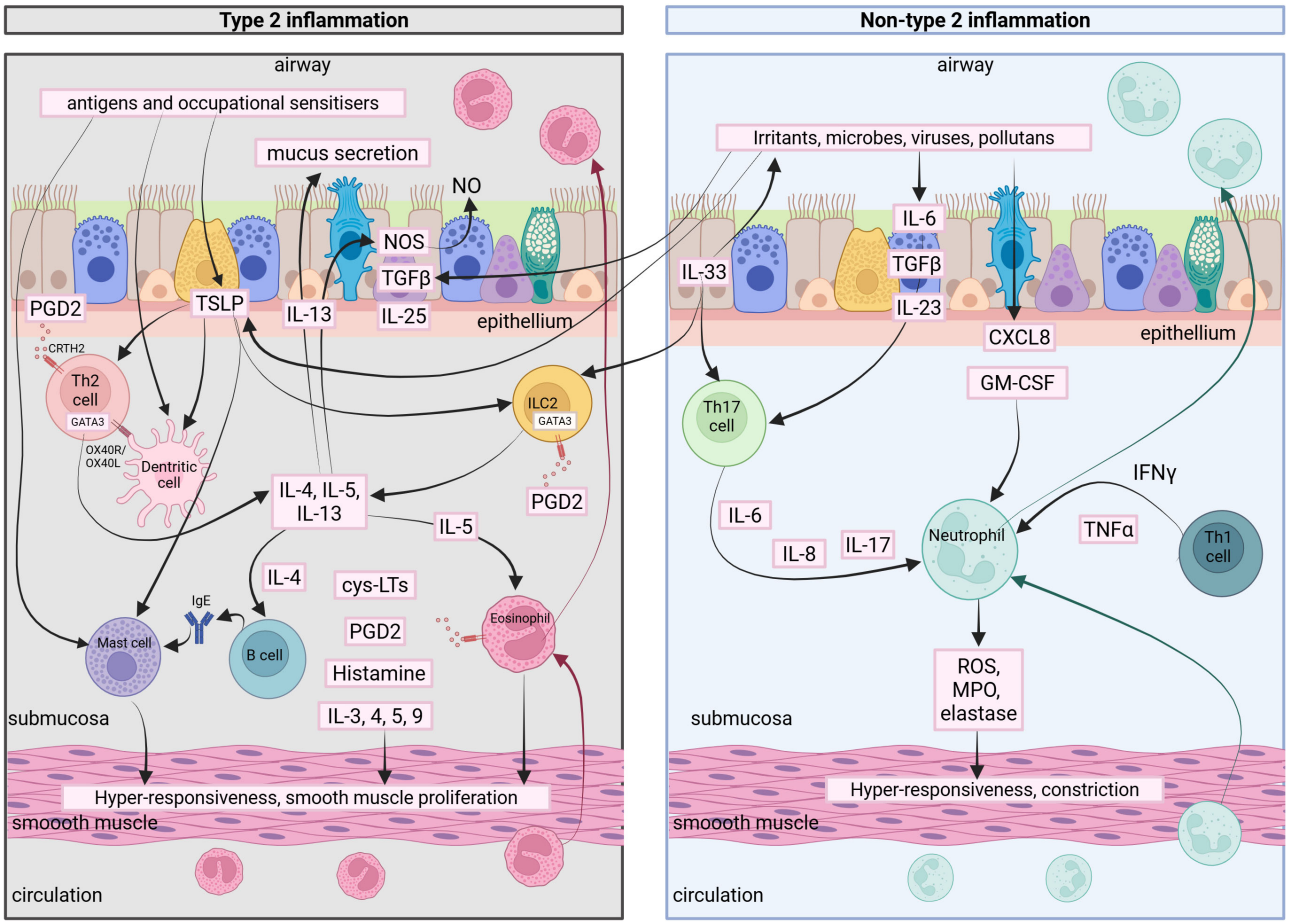
Pathomechanism of type 2 (T2) and non–type 2 (non−T2) inflammation. Schematic representation of inflammatory pathways in the airway epithelium. T2 inflammation is driven by Th2 cells and ILC2s, with key roles of IL−4, IL−5, and IL−13, leading to IgE production, eosinophilic inflammation, mucus hypersecretion, and airway hyperresponsiveness. In contrast, non−T2 inflammation is mediated by Th1 and Th17 responses, involving cytokines such as IL−6, IL−8, IL−17, IFN−γ, and TNF−α, and is characterized by neutrophilic inflammation, epithelial activation and airway constriction. Figure was created with bioRender.com. cys-LTs, cysteinyl leukotrienes; GATA3, GATA binding protein 3; GM-CSF, granulocyte-macrophage colony-stimulating factor; IFN, interferon; ILC2, group 2 innate lymphoid cells; MPO, myeloperoxidase; NO, nitric oxide; NOS, nitric oxide synthase; PGD2, prostaglandin D2; ROS, reactive oxygen species; TGF-β, transforming growth factor beta; Th, T helper cells; TNF, tumor necrosis factor; TSLP, thymic stromal lymphopoietin.

Beyond abnormalities in lipid mediator pathways, N−ERD is associated with chronic, extensive type 2 eosinophilic inflammation, increased Th2 cytokine expression, and intricate interactions among mast cells, basophils, epithelial cells, platelets, and other components of the innate immune system (1–4, 7). In particular, the upper airway mucosa in N−ERD demonstrates marked hyperplasia and activation of mast cells and eosinophils (3). The resulting inflammatory milieu is heterogeneous and mixed, with a predominance of type 2 cytokines (IL−4, IL−5, IL−13) and encompasses both eosinophilic and non−eosinophilic asthma phenotypes (3). Type 2 cytokines, particularly IL-4 and IL-13, contribute to barrier disruption by downregulating and disorganizing tight junction proteins, while also promoting basal cell hyperplasia, see **Figure 2** (4). Severe type 2 inflammation involving both the upper and lower airways is a hallmark of N-ERD and is thought to contribute substantially to the marked clinical severity of asthma and CRSwNP observed in these patients (7).

Especially, macrophages undergo proinflammatory metabolic and epigenetic reprogramming, accompanied by M2 macrophage polarization and disturbances in acylcarnitine metabolism, which may drive type 2 inflammation (8–10). Elevated acylcarnitine levels and an exaggerated macrophage response to inflammatory stimuli, marked by increased production of lipid mediators, chemokines, and cytokines, highlight potential targets for biologic therapies aimed at modulating these dysregulated pathways (8–10). In addition, the terminal metabolite of 15−LOX−1, 15-oxo-eicosatetraenoic acid (15-oxo-ETE) may represent a promising therapeutic target. Increased levels of this metabolite have been observed in nasal polyps tissue, plasma and induced sputum in patients with N−ERD, while its elevated plasma level indicate a severe disease endotype of N-ERD (11–15).

Emerging evidence further suggests that airway inflammation in N−ERD is heterogeneous and may extend beyond the classical type 2 inflammatory paradigm, affecting both upper and lower airways (1–3, 7).

## 2 Biologic therapies

Several biologic therapies are currently available for the management of severe asthma and related inflammatory airway diseases, many of which target key pathways of type 2 inflammation. Appropriate treatment selection in the N−ERD population is crucial due to the higher prevalence of severe asthma and a greater frequency of revision sinus surgeries in the course of CRSwNP compared with patients with ATA (5, 16).

In patients with CRSwNP receiving active treatment (including intranasal corticosteroids), almost 24% will undergo endoscopic sinus surgery (ESS) in 10 years and 20% of them will need a revision surgery within next 5 years (17). While in N-ERD group, 80% of patients required additional surgery after 2 years caused by regrowth of nasal polyps (18). Similarly in asthma, subjects with N-ERD more often have severe disease and before the era of biologics substantial proportion of these subjects were treated with oral corticosteroids (5).

**Figure 3:**
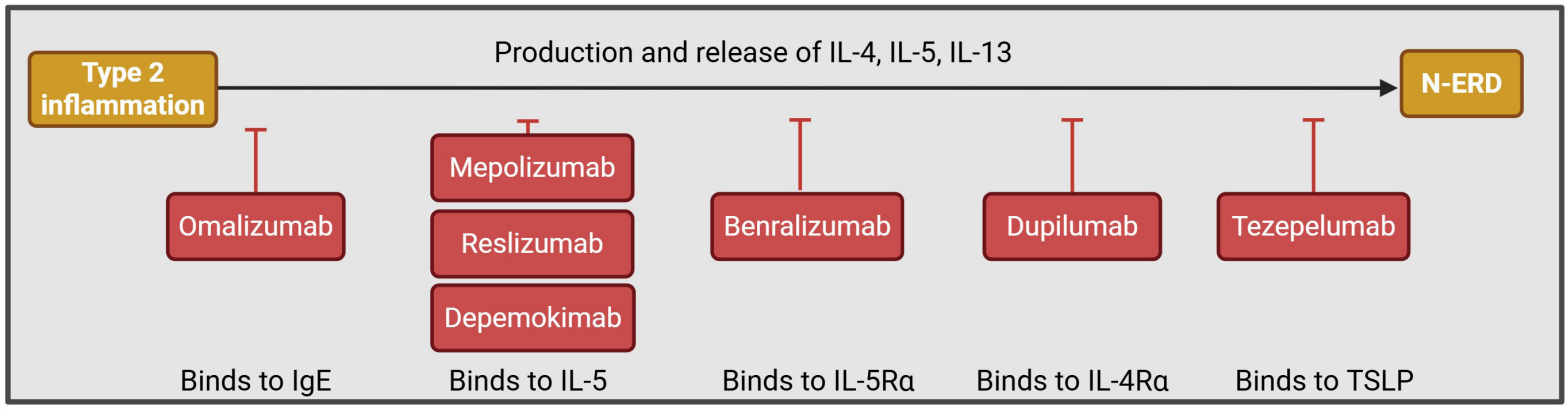
Mechanisms of action of biologic therapies targeting type 2 inflammation in N−ERD. Schematic illustration of the key pathways involved in type 2 inflammation, including IL−4, IL−5 and IL−13 signaling, and the corresponding targets of biologic therapies. Omalizumab inhibits IgE binding to high−affinity receptors; mepolizumab and reslizumab target IL−5; benralizumab binds IL−5 receptor α (IL−5Rα); dupilumab blocks IL−4 receptor α (IL−4Rα), inhibiting IL−4 and IL−13 signaling; and tezepelumab targets thymic stromal lymphopoietin (TSLP).

Therefore, the future of N-ERD management is increasingly focused on targeted biologic therapies that interfere with key inflammatory pathways. Mepolizumab, reslizumab, and benralizumab inhibit the interleukin−5 (IL−5) pathway by targeting IL−5 or its receptor, while dupilumab blocks IL−4 and IL−13 signaling through antagonism of the IL−4 receptor−α (19). Tezepelumab acts upstream by inhibiting thymic stromal lymphopoietin (TSLP), thereby preventing initiation of the epithelial−driven inflammatory cascade (19), see **Figure 3**.

### 2.1 Omalizumab

Omalizumab is a humanized IgG1 monoclonal antibody that binds circulating IgE, thereby preventing its interaction with the high−affinity IgE receptor (FcϵRI), leading to reduced mast cell activation and rapid downregulation of FcϵRI expression on mast cells, basophils and dendritic cells (1, 20).

The efficacy of omalizumab in N−ERD has been demonstrated in several clinical trials and real−world studies, although it should be noted that not all studies included placebo−controlled groups. Overall, treatment is consistently associated with a reduction in nasal polyp score (NPS) and improvements in lung function (20). These changes are accompanied by decreased mast cell activation, reduction in peripheral blood eosinophil counts, and lower levels of urinary eicosanoid metabolites such as leukotriene E4 (LTE4) and tetranor−PGDM, indicating a broad anti-inflammatory effect on both upper and lower airways (20). Omalizumab has also been shown to reduce exacerbation rates, hospitalizations and the need for systemic corticosteroids in patients with N−ERD, highlighting its beneficial impact on overall disease burden (21, 22).

Importantly, similar improvements in NPS have been observed in both N-ERD and ATA with CRSwNP populations after approximately 24 weeks of therapy, suggesting that omalizumab is effective irrespective of aspirin sensitivity status (23). However, patients with N-ERD may still represent a more treatment refractory subgroup (23).

>A distinctive and clinically relevant effect of omalizumab in N−ERD is its potential to increase tolerance to non−steroidal anti−inflammatory drugs (NSAIDs), including aspirin (24–26). In this context, “aspirin tolerance” refers to a reduced likelihood or severity of respiratory reactions (e.g. bronchospasm, nasal congestion, rhinorrhea) during controlled oral aspirin challenge.

Omalizumab therapy has been associated with the development of aspirin tolerance in approximately 56–71% of patients with N−ERD, indicating that a substantial proportion can achieve reduced or absent hypersensitivity reactions during aspirin exposure (24, 25, 27). This is particularly important in the context of aspirin desensitization, a therapeutic procedure in which gradually increasing doses of aspirin are administered under medical supervision until a maintenance dose is tolerated, allowing long−term treatment (1). However, not all studies demonstrate consistent benefits. For example, some reports found no significant differences in reaction severity during aspirin desensitization between patients treated with omalizumab and untreated controls, indicating that the effect on clinical reactivity may not be universal (28).

At the immunological level, omalizumab treatment is associated with an increase in total serum IgE, reflecting the formation of IgE–anti−IgE complexes, alongside reductions in eosinophilic inflammation markers such as eosinophilic cationic protein and relative blood eosinophilia (27). In addition, among atopic individuals, tissue IgE levels and local eosinophil counts within nasal polyps have been shown to decrease significantly after short−term therapy, supporting a local anti−inflammatory effect (27).

Overall, omalizumab demonstrates consistent clinical and immunologic benefits in patients with N−ERD, including reductions in nasal polyp burden, suppression of key inflammatory mediators (e.g. LTE4, PGD2 metabolites), and improvements in respiratory function. It is also associated with increased aspirin challenge thresholds and higher rates of NSAID tolerance, although its effects on aspirin desensitization outcomes remain variable across studies.

### 2.2 Anti-IL 5 and anti-IL 5Rα

Mepolizumab, reslizumab and depemokimab are humanized monoclonal antibodies targeting IL−5 (IgG1κ and IgG4κ, respectively), while benralizumab is a humanized IgG1κ antibody that inhibits IL−5 signaling by binding to IL−5Rα leading to antibody−dependent cellular cytotoxicity and near−complete depletion of eosinophils (1, 19, 29, 30). Depemokimab is an ultra-long-acting biologic with a prolonged half-life, enabling sustained suppression of type 2 inflammation with twice−yearly dosing in patients with asthma and/or CRSwNP (31).

Mepolizumab, reslizumab, and benralizumab have been shown to reduce asthma exacerbations (1, 19, 29). In addition, mepolizumab has demonstrated beneficial effects on airway remodeling, including reductions in airway smooth muscle mass, epithelial damage and tissue eosinophilia, suggesting potential structural modifying properties in asthmatic airway tissue (32).

Mepolizumab treatment has been associated with significant improvements in patient−reported outcomes, including reductions in Sino-Nasal Outcome Test-22 (SNOT−22), as well as better asthma control and improvement in key symptoms such as anosmia and nasal obstruction (33).

At the molecular level, mepolizumab exerts broader immunomodulatory effects beyond eosinophil depletion. In N−ERD patients, treatment leads to reduced production of proinflammatory eicosanoids, including urinary LTE4 and prostaglandin D2 metabolites, as well as nasal prostanoids (PGF2α, tetranor−PGFM), leukotriene B4 (LTB4), and thromboxane B2 (TXB2) (34). These changes reflect suppression of key pathways involved in arachidonic acid metabolism, which are central to N−ERD pathophysiology.

Interestingly, despite the reduction in eosinophil numbers, residual circulating eosinophils and basophils show increased surface expression of CRTH2 (chemoattractant receptor−homologous molecule expressed on TH2 cells; also known as prostaglandin D2 receptor 2, DP2), a receptor involved in type 2 cell trafficking and activation (34). This may indicate a compensatory mechanism or persistent activation of type 2 pathways despite eosinophil depletion.

Transcriptomic analyses further demonstrate that mepolizumab influences epithelial barrier function in N−ERD. Treatment is associated with upregulation of genes involved in tight junction formation (e.g. *TJP3, ACTN4, AMOT*) and ciliary structure, alongside downregulation of genes linked to epithelial dysfunction (34). These findings suggest that anti−IL−5 therapy may partially restore epithelial integrity in addition to suppressing inflammation.

Single−cell RNA sequencing studies have also shown that IL-5Rα expression is not restricted to eosinophils in N−ERD but is present in multiple cell populations, including plasma cells, mast cells, and ciliated epithelial cells (34). This broader expression pattern may help explain the complex and sometimes incomplete clinical responses to IL−5 targeted therapies.

Clinical trial data further supports the efficacy of this pathway in CRSwNP. In the SYNAPSE study, mepolizumab significantly reduced nasal polyp size, nasal obstruction, and the need for systemic corticosteroids and surgery in patients with severe CRSwNP, including those with N−ERD (35). Similarly, in the OSTRO trial, benralizumab reduced nasal polyp score, nasal obstruction, and impairment of smell compared with placebo (36).

The ANCHOR−1 and ANCHOR−2 trials included patients with N−ERD and demonstrated that treatment with depemokimab resulted in improvements in nasal polyp score and nasal obstruction, as measured by the verbal rating scale (30). In the SWIFT−1 and SWIFT−2 trials, depemokimab significantly reduced the annualized exacerbation rate in patients with severe eosinophilic asthma (37). Type 2 asthma with coexisting CRSwNP represents a distinct clinical phenotype that is likely to derive enhanced benefit from depemokimab therapy (38, 39).

Despite these beneficial effects, responses to anti−IL−5/IL−5Rα therapies in N−ERD are heterogeneous. Some studies report no significant improvement in lung function despite reductions in inflammation, suggesting dissociation between biomarker changes and clinical outcomes (40, 41). Moreover, relatively high rates of treatment discontinuation have been observed, indicating suboptimal response in a subset of patients (42).

### 2.3 Dupilumab

Dupilumab is a humanized IgG4 monoclonal antibody that targets the IL−4 receptor α (IL−4Rα) subunit shared by IL−4 and IL−13 (43). IL−4Rα is broadly expressed on multiple cell types involved in type 2 inflammation, including immune cells such as B cells, dendritic cells, and T cells, as well as non−hematopoietic cells including lung epithelial cells, stromal cells, and macrophages (44).

In the pivotal SINUS−24 and SINUS−52 trials, dupilumab significantly improved NPS, nasal congestion and Lund-Mackay score in patients with CRSwNP, including those with N−ERD, confirming its efficacy across this difficult−to−treat population (43).

Furthermore, dupilumab has been shown to act as an adjunct to ESS, delaying postoperative recurrence of nasal polyps and improving long−term disease control (45).

Beyond clinical efficacy, dupilumab exerts profound effects on local inflammatory pathways in N−ERD. Treatment leads to reduced nasal expression of key mediators such as oncostatin M (OSM), IL−4, IL−6, and colony−stimulating factors (CSF−1, CSF−3), which are involved in epithelial barrier dysfunction and immune activation (46). Mechanistically, dupilumab may act (1) directly, by blocking IL-4Rα signaling on IL-6–producing cells, including epithelial cells and macrophages and (2) indirectly, by reducing OSM levels, which in turn decreases IL-6 production by fibroblasts and other structural cells, contributing to restoration of epithelial integrity (46).

In addition, dupilumab appears to normalize dysregulated eicosanoid metabolism characteristic of N−ERD (47). It has been proposed that treatment restores prostaglandin E2 (PGE2) signaling by upregulating COX-2 and PGE2 receptors (EP2) receptor expression in airway epithelial cells and possibly macrophages, as well as increasing expression of membrane bound prostaglandin E synthase-1 (mPGES-1) (47). This results in increased PGE2 production, which counterbalances leukotriene overproduction and improves airway inflammation and obstruction (47).

Clinically, dupilumab induces rapid and robust responses in patients with N−ERD. Improvements have been observed as early as 1 month after treatment initiation and include reductions in NPS, SNOT−22 and Lund-Mackay score, as well as improvements in sense of smell, asthma control (ACT), quality of life (AQLQ), and lung function (48–50). These changes are accompanied by decreases in biomarkers of type 2 inflammation, including nasal and urinary LTE4 and fractional exhaled nitric oxide (FeNO), alongside increased levels of anti−inflammatory PGE2 (48–50). Additionally, patients with N-ERD presented significant reduction in Lund Mackay score from mean 21.5 to 4 points after 6 months of dupilumab treatment (49).

Transcriptomic analyses further support the broad immunomodulatory effects of dupilumab (48). RNA sequencing of inferior turbinate samples has shown downregulation of multiple pathways related to type 2 inflammation, cytokine signaling and epithelial dysfunction, with a smaller number of pathways upregulated, primarily associated with epithelial repair and barrier restoration (48).

At the immunological level, dupilumab treatment is associated with a reduction in total IgE levels in both serum and nasal secretions, reflecting inhibition of IL−4/IL−13–driven class switching in B cells (48). In contrast, no significant changes have been observed in IgA, total IgG, or IgG4 levels, and peripheral blood eosinophil counts may remain stable or even transiently increase, reflecting redistribution rather than ongoing tissue inflammation (48).

Interestingly, the effects of dupilumab on the nasal microbiome remain inconsistent. Nasal microbiome during dupilumab treatment did not change diversity or composition (51). While in another study, dupilumab therapy was associated with a shift in the nasal microbiota toward a profile resembling that of healthy controls, while the gastrointestinal microbiota remained unchanged (52).

### 2.4 Tezepelumab

Tezepelumab is a human IgG2λ monoclonal antibody that targets thymic stromal lymphopoietin (TSLP), preventing its interaction with the heterodimeric TSLP receptor (TSLPR) and thereby inhibiting downstream signaling; it is approved for the treatment of severe asthma without phenotype or biomarker restrictions (1, 19). TSLP is a four α-helical type I cytokine produced by epithelial and stromal cells in the lungs, but can be produce also by mast cells, dendritic cells (DCs) and basophils (53). TSLP binds with TSLPR expressed on DCs, mast cells, macrophages, basophils, group 2 innate lymphoid cells (ILC2), T cells as well as epithelial cells (53).

Tezepelumab is currently approved for the treatment of severe asthma without phenotype or biomarker restrictions, reflecting its upstream mechanism of action. However, evidence for its use in N-ERD remains limited and is largely derived from subgroup analyses and mechanistic studies.

At the mechanistic level, TSLP is highly expressed in nasal polyp tissue from patients with N-ERD, supporting its central role in disease pathogenesis (54, 55). Increased expression of TSLP has been observed particularly in airway epithelial and basal cells, where it is closely linked to epithelial dysfunction and activation of downstream inflammatory cascades (55). Notably, basal cell expression of TSLP (as well as IL-33) correlates with transcription factors involved in basal cell differentiation, suggesting that epithelial cell state may directly influence inflammatory potential in N-ERD (54, 55).

In addition to tissue findings, increased expression of TSLP and its receptor (TSLPR) has been reported in peripheral blood cells from asthmatics patients with N-ERD compared with healthy controls, although similar levels may be observed in patients with CRSwNP irrespective of aspirin sensitivity (56). Likewise, nasal tissue analyses demonstrate elevated TSLP expression in N-ERD compared with healthy controls, whereas TSLPR expression does not appear to differ significantly (56). Peripheral blood mRNA levels of TSLP/TSLPR should be investigated as potential new minimally invasive biomarkers that could assist in selecting patients for treatment with specific antagonists (56). So, probably these overexpression of *TSLP* and *TSLPR* are common with advanced pathological changes in sinuses rather than unique for N-ERD.

In the NAVIGATOR trial, which evaluated tezepelumab in patients with severe, uncontrolled asthma, individuals with N-ERD experienced some of the greatest reductions in annualized asthma exacerbation rates (AAER) compared with placebo (57). Additionally, within this subgroup, tezepelumab was associated with improvements in lung function, asthma control, rhinosinusitis symptoms and health related quality of life (58). These findings suggest that targeting TSLP may be particularly beneficial in patients with N-ERD, who are characterized by severe, multi-compartment type 2 inflammation.

Emerging data also indicate potential benefits of tezepelumab in upper airway disease. Treatment has been associated with reductions in SNOT-22 scores, NPS and the need for oral corticosteroids, suggesting clinically meaningful effects on CRSwNP manifestations in N-ERD (59).

Overall, tezepelumab represents a promising upstream therapeutic strategy in N−ERD by targeting epithelial−derived inflammatory signaling that may contribute to both upper and lower airway disease. However, given the currently limited and largely indirect evidence in N−ERD, further dedicated studies are needed to define its clinical efficacy, optimal patient selection, and positioning relative to other biologic therapies in this population.

## 3 Comparison

Given the heterogeneity of N−ERD and the availability of multiple biologic therapies targeting distinct inflammatory pathways, individualized treatment selection remains a key challenge in clinical practice.

Comparative analyses of biologic therapies (omalizumab, mepolizumab, benralizumab and dupilumab) have shown that the aspirin dose required to elicit respiratory symptoms during an oral aspirin challenge increases significantly only in patients treated with omalizumab or dupilumab, but not in those receiving mepolizumab or benralizumab (60). This finding translates into higher rates of NSAID tolerance, defined as the ability to tolerate aspirin, without clinically relevant respiratory reactions, which were observed in 60% of patients treated with omalizumab and 40% of those treated with dupilumab, compared with 22% in patients receiving mepolizumab or benralizumab (60). Overall, biologic therapies (including dupilumab, omalizumab, mepolizumab, benralizumab) are associated with meaningful clinical benefits in patients with CRSwNP and comorbid N−ERD (61).

However, comparative effectiveness studies consistently suggest differences in the magnitude of response among these agents. In the treatment of CRSwNP, dupilumab demonstrated moderate superiority in both objective and patient−reported outcomes, as well as a greater reduction in the need for rescue surgery, followed by omalizumab and mepolizumab (62).

Direct head−to−head comparisons in N−ERD further support this hierarchy. Dupilumab was associated with significantly greater improvements than omalizumab in NPS, loss of smell (LoS), total symptom score (TSS), University of Pennsylvania Smell Identification Test (UPSIT), and nasal congestion scores (63). Consistently, the EVEREST trial reported significantly greater improvements in NPS and UPSIT with dupilumab compared with omalizumab (64). Another study confirmed that dupilumab produced significantly greater reductions in SNOT−22 scores in patients with N−ERD compared with mepolizumab, reslizumab, benralizumab, and omalizumab (42).

Moreover, dupilumab demonstrated significant efficacy in improving CRSwNP outcomes in both aspirin−tolerant patients and those with N−ERD, with markedly greater benefits observed in the N−ERD subgroup, underscoring the particular sensitivity of this phenotype to IL−4/IL−13 pathway blockade (65). Importantly, patients with N−ERD who showed inadequate responses to anti−IL−5/anti−IL−5Rα therapies have been reported to respond favorably to dupilumab, including reductions in asthma exacerbation rates and improved overall disease control (66). These findings are supported by a meta−analysis (including omalizumab, anti-IL-5/anti-IL-5Rα, dupilumab) showing that dupilumab provides the greatest overall benefit in patients with N−ERD (67).

In contrast, data on tezepelumab in N−ERD remain limited. In the broader CRSwNP population, meta−analyses suggest that tezepelumab and dupilumab achieve comparable improvements in NPS and UPSIT, whereas dupilumab appears more effective in reducing overall disease severity as assessed by the Lund-Mackay score (68). Tezepelumab improved NPS and nasal congestion, demonstrating efficacy in both T2−high and T2−low patients stratified by blood eosinophil counts (≥300 cells/μL and <150 cells/μL) (69). In contrast, depemokimab appeared to be effective primarily in T2−high patients (≥220 cells/μL) and showed less pronounced improvements in Lund–Mackay score and NPS compared with tezepelumab (69).

From a therapeutic sequencing and health−economic perspective, aspirin desensitization following ESS, or the use of dupilumab as salvage therapy after failed desensitization, has been shown to be more cost−effective than upfront dupilumab therapy (70). Ideally, aspirin therapy after desensitization (ATAD) should be initiated within 1–2 months after nasal polyp debulking, as ATAD is more effective in preventing polyp regrowth than in reversing established CRSwNP (1).

Notably, dupilumab reduced sinonasal eosinophilic inflammation only in patients receiving concomitant ATAD, whereas patients treated with dupilumab alone showed no significant reduction in local eosinophilia but developed increased peripheral blood eosinophil counts (71). This observation suggests that ATAD may facilitate effective suppression of tissue-level eosinophilic inflammation, while blood eosinophilia during dupilumab therapy likely reflects redistribution of eosinophils rather than persistent local inflammatory activity.

The introduction of biologic therapies has therefore challenged the traditional role of ATAD in N−ERD management, highlighting the need for comparative efficacy data and phenotype−based treatment strategies (72, 73). There is also some evidence suggesting a synergistic effect between ATAD and biologic therapies in severe N-ERD patients, although these data remain limited and require confirmation in larger cohorts (71, 72, 74). Retrospective analyses indicate that while all treatment modalities provide specific benefits, combination approaches yield the most comprehensive outcomes, supporting a phenotype−guided strategy with ATAD for milder disease, biologic monotherapy for moderate disease, and combined therapy for severe N−ERD phenotypes (72). All publications, including the number of patients with N−ERD and the therapeutic effect of each biologic drug, are presented in **Table 1**. These studies differ substantially in study design, which significantly limits the validity of indirect comparisons (which always should be interpreted with caution) between biologic therapies.

**Table 1:** Summary of clinical studies evaluating biologic therapies in patients with N−ERD.

Study	Biologic	Study design	N-ERD patients	Outcomes
Hayashi et al. (22) 2016	omalizumab	open label trial with no control group	21	↓ exacerbations, hospitalizations, daily doses of systemic corticosteroid, ↓ nasal and asthma related symptom scores, ↓ urinary LTE4, PGD2M, ↓ peripheral blood eosinophils
Hayashi et al. (24) 2020	omalizumab	randomized controlled trial	16	↓ symptoms during an oral aspirin challenge, resulting in aspirin tolerance in 62.5%, ↓ urinary LTE4
Gevaert et al. (23) 2022	omalizumab	randomized controlled trial	72	↓ NPS but without differences between N-ERD and ATA
Lang et al. (25) 2018	omalizumab	randomized controlled trial	12	↓ symptoms during an oral aspirin challenge, ↑ aspirin tolerance in 71%
Forster-Ruhrmann et al. (82) 2020	omalizumab	retrospective cohort study	16	↓ NPS, ↓ CRS symptoms, ↑ ACT, ↑ FEV1%
Lee et al. (83) 2018	omalizumab	retrospective cohort study	26	78.9% of the patients responded to the therapy
Armengot-Carceller et al. (84) 2021	omalizumab	case series	19	↓ NP size, ↑ QoL, no differences in improvement between N-ERD and ATA
Tiotiu et al. (85) 2020	omalizumab	case series	9	better reduction of NP in N-ERD compared to ATA
Jean et al. (21) 2019	omalizumab	case series	29	↓ the use of corticosteroids and SABAs
Phillips-Angles et al. (26) 2017	omalizumab	observational study	7	patients achieved aspirin tolerance
Waldram et al. (28) 2018	omalizumab	retrospective cohort study	8	no differences in reaction type or severity with omalizumab to aspirin
Quint et al. (27) 2022	omalizumab	open label trial with no control group	33	56% developed complete aspirin tolerance, ↑ total serum IgE, ↓ tissue IgE, ↓ eosinophilic cationic protein, ↓ blood eosinophilia, ↓ local eosinophils only in atopic individuals
Jorge Sánchez et al. (60) 2023	omalizumab, benralizumab, dupilumab, mepolizumab	head−to−head randomized controlled trial	38	only omalizumab and dupilumab led to aspirin tolerance (but not mepolizumab or benralizumab)
Buchheit et al. (34) 2021	mepolizumab	open label trial with no control group	18	↓ nasal PGF2α, PGD2, LTB4, TXB, ↓ urinary t-PGD2M, LTE4,
Tuttle et al. (33) 2018	mepolizumab	case series	22	↑ ACT, ↓ blood eosinophils, ↓SNOT-22
Bachert et al. (35) 2022	mepolizumab	randomized controlled trial	108	↓ nasal polyps and nasal obstruction in CRSwNP regardless of the presence of comorbid N-ERD
Bachert et al. (36) 2022	benralizumab	randomized controlled trial	121	↓ NPS, ↓ nasal obstruction
Tversky et al. (86) 2021	benralizumab	randomized controlled trial	11	↓ NPS, ↓ SNOT-22
Numata et al. (40) 2020	benralizumab	case series	7	no significant difference in the %FEV1
Wangberg et al. (42) 2022	omalizumab mepolizumab reslizumab benralizumab dupilumab	retrospective cohort study	74	↓ SNOT-22 on dupilumab
Lyly et al. (87) 2025	mepolizumab	randomized controlled trial	120	ongoing study
Suikkila et al. (61) 2025	omalizumab mepolizumab benralizumab dupilumab	retrospective cohort study	29	↓ NPS, ↓ SNOT-22, ↓ otologic symptoms
Gevaert et al. (30) 2025	depemokimab	randomized controlled trial	85	↓ NPS, ↓ nasal obstruction
Bartosik et al. (51) 2025	dupilumab	open label trial with no control group	28	↓ NPS, ↓ SNOT-22, no changes in nasal microbiome
Ryser et al. (52) 2025	dupilumab	prospective cohort study	10	nasal microbiota normalization, no changes in gut microbiome
De Corso et al. (64) 2025	omalizumab dupilumab	head−to−head randomized controlled trial	145	↓ NPS on dupilumab, ↑UPSIT on dupilumab
Bachert et al. (43) 2019	dupilumab	randomized controlled trial	204	↓ NPS, ↓ nasal congestion, ↓ LM score, ↑ lung function
Buchheit et al. (71) 2023	dupilumab + ATAD	prospective cohort study	22	synergism between biologic treatment with ATAD
Buchheit et al. (48) 2022	dupilumab	open label trial with no control group	22	↓ NPS, ↓ SNOT-22, ↓ urinary and nasal LTE4, ↓ serum and nasal IgE, ↑ lung function
Laidlaw et al. (88) 2019	dupilumab	observational study	19	↓ SNOT-22, ↑ asthma control, ↑ lung function
Bavaro et al. (66) 2021	dupilumab	retrospective cohort study	41	↓ SNOT-22, ↑ asthma control, ↑ lung function, ↓ exacerbations
Schneider et al. (89) 2023	dupilumab	open label trial with no control group	31	patients achieved aspirin tolerance, ↓ SNOT-22, ↑ACT, ↑lung function, ↓ urinary LTE4 ↓serum and nasal IgE
Mustafa et al. (49) 2021	dupilumab	open label trial with no control group	5	increased aspirin tolerance
Patel et al. (45) 2022	dupilumab	case series	8	adjunct to ESS
Oykhman et al. (67) 2022	omalizumab mepolizumab benralizumab reslizumab dupilumab	network meta−analysis	meta-analysis	dupilumab with the greatest clinical benefits
Laidlaw et al. (58) 2025	tezepelumab	post-hoc analysis of subgroup from randomized controlled trial	43	↓ SNOT-22 ↑ ACT ↑lung function, ↑ quality of life
Carr et al. (57) 2024	tezepelumab	subgroup analysis of randomized controlled trial	43	↓ asthma exacerbations

↓, decreased; ↑, increased.

Substantial heterogeneity across studies further limits the comparability of reported outcomes. Although some biologic agents, particularly dupilumab, appear to demonstrate robust clinical effects, the lack of adequately powered head−to−head randomized trials in N−ERD and reliance on indirect comparisons preclude definitive conclusions regarding comparative efficacy.

## 4 Personalized medicine in N−ERD: how to individualize biologic therapy

N−ERD is a clinically and biologically heterogeneous disease, encompassing variable degrees of upper and lower airway involvement, differences in inflammatory endotypes and distinct responses to pharmacological and surgical interventions. With the increasing availability of biologic therapies targeting different inflammatory pathways, a personalized approach to treatment selection has become essential to optimize outcomes and avoid ineffective therapy.

Importantly, no single biologic is universally effective for all patients, and treatment decisions should reflect the predominant drivers of disease in a given individual.

Blood eosinophil count and fractional exhaled nitric oxide (FeNO) are established biomarkers predicting both the likelihood and magnitude of response to asthma biologic therapies (1, 19). Higher baseline blood eosinophil levels and elevated FeNO are associated with greater reductions in asthma exacerbations, particularly for dupilumab and tezepelumab, whereas this relationship is less pronounced for anti−IL−5/IL−5R therapies (1, 19). Available evidence suggests that the highest therapeutic benefit from biologic treatment is observed in patients with baseline blood eosinophil levels ≥300 cells/µL (19). In N-ERD patients with bilateral nasal polyps who have undergone ESS, biologic treatment is indicated including evidence of type 2 inflammation defined by a blood eosinophil count ≥150 cells/µL, total IgE ≥100 IU/mL or tissue eosinophilia ≥10 eosinophils per high-power field (1).

Regular clinical reassessment of patients receiving biologic therapies is essential, as the development of anti−drug antibodies (ADAs), neutralizing antibodies, may adversely affect treatment efficacy and safety. Although the overall incidence of treatment−emergent ADAs is low (<3%), higher rates have been reported with certain agents, particularly benralizumab (8.35%) and dupilumab (7.61%), compared with lower incidences observed with mepolizumab (3.63%), reslizumab (4.39%), tezepelumab (1.12%), and omalizumab (<1%) (75). Moreover, neutralizing antibodies occur most frequently with benralizumab (up to 7.12%), especially with longer dosing intervals, underscoring the need for ongoing monitoring and timely re−evaluation in patients with loss of clinical response (75).

**Figure 4:**
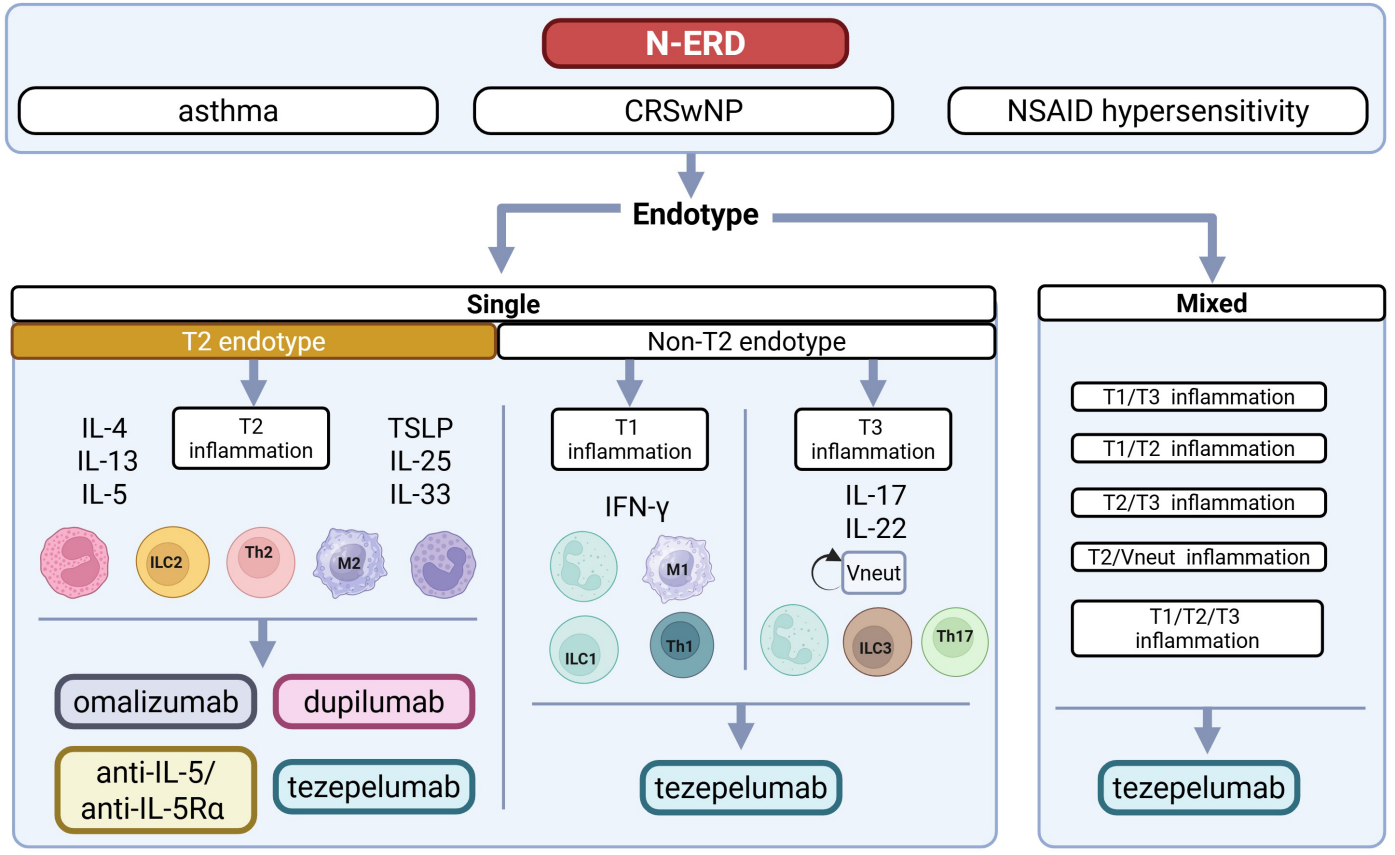
Heterogeneity of endotypes in NSAID-exacerbated respiratory disease (N−ERD). N−ERD can be classified into single (T2 or non−T2) or mixed inflammatory endotypes. The T2 endotype is driven by IL−4, IL−5, and IL−13, with involvement of eosinophils, ILC2s, Th2, macrophages M2 and basophils. Non−T2 endotypes include T1 inflammation (mediated by IFN−γ with neutrophils, macrophages M1, ILC1s, Th1) and T3 inflammation (associated with IL−17, IL−22 with neutrophils, ILC3s and Th17, including neutrophil variant, Vneut). Mixed endotypes reflect overlapping inflammatory patterns (T1/T2, T2/T3, T1/T3, or combined T1/T2/T3 responses). The diagram also illustrates current biologic therapies targeting these pathways, including anti−IgE, anti−IL−5/IL−5Rα, anti−IL−4Rα, and anti−TSLP strategies. Figure created with BioRender.com. CRSwNP, chronic rhinosinusitis with nasal polyps; IFN, interferon; ILC, innate lymphoid cells; ILC1/2/3, group 1/2/3 innate lymphoid cells; IL, interleukin; T1, type 1 inflammation; T2, type 2 inflammation; T3, type 3 inflammation; Th, T helper cells; TSLP, thymic stromal lymphopoietin.

Although N−ERD is relatively uniform in its lipid mediator profile, it demonstrates substantial variation in type 1, type 2, and type 3 cytokine expression. This heterogeneity may affect how patients respond to therapies targeting type 2 inflammation. In general, N−ERD is characterized by a complex and diverse inflammatory milieu with fluctuating cytokine levels. Current evidence indicates that approximately half of patients with N−ERD exhibit a nonclassical spectrum of type 2 inflammation (76–81), see **Figure 4**.

Biomarkers such as AA metabolites (e.g. LTE4, PGD2, 15-oxo-ETE), eosinophil counts and transcriptomic signatures show promise, yet their clinical utility is currently limited by issues with reproducibility, lack of standardization and uncertain applicability in routine decision−making for biologic selection. Future research directions in N−ERD are increasingly focused on biomarker−guided therapeutic strategies, integration of multi−omics approaches, and the use of artificial intelligence−based predictive models to improve patient stratification. Additionally, well−designed comparative effectiveness studies and the development of personalized treatment algorithms will be essential to optimize biologic selection and long−term disease management.

## 5 Conclusion

N−ERD is a complex and severe inflammatory airway disease characterized by dysregulated eicosanoid metabolism and pronounced type 2 inflammation involving both the upper and lower airways. The advent of biologic therapies targeting key inflammatory pathways has substantially expanded treatment options for affected patients. Omalizumab, anti−IL−5/IL−5Rα agents, dupilumab and tezepelumab have all demonstrated clinical benefit in N−ERD, although their efficacy varies across sinonasal, pulmonary, and patient−reported outcomes. Current evidence consistently indicates that dupilumab provides the most comprehensive improvements across these disease domains, particularly in patients who are refractory to other biologic therapies, while data on tezepelumab remains limited but encouraging, especially with respect to exacerbation reduction.

Optimal management of N−ERD requires an individualized, multidisciplinary approach integrating pharmacotherapy, endoscopic sinus surgery and/or aspirin desensitization. Importantly, the combination of biologic therapy with aspirin therapy may confer additive or synergistic benefits through complementary mechanisms of action. Given the high cost of biologic therapies, treatment decisions should incorporate cost-effectiveness, optimal patients selection, and sequencing strategies, while also considering long-term healthcare sustainability.

Finally, well−designed head−to−head clinical trials and biomarker−driven studies are needed to refine treatment selection, improve patient stratification, and better define long−term disease−modifying strategies in N−ERD.
